# High-Flow Nasal Cannula versus Noninvasive Ventilation in AECOPD Patients with Respiratory Acidosis: A Retrospective Propensity Score-Matched Study

**DOI:** 10.1155/2023/6377441

**Published:** 2023-04-15

**Authors:** Meng Wang, Feifan Zhao, Lina Sun, Ying Liang, Wei Yan, Xiaoyan Sun, Qingtao Zhou, Bei He

**Affiliations:** Department of Respiratory and Critical Care Medicine, Peking University Third Hospital, Beijing 100191, China

## Abstract

**Background:**

Limited data are available about the clinical outcomes of AECOPD patients with respiratory acidosis treated with HFNC versus NIV.

**Methods:**

We conducted a retrospective study to compare the efficacy of HFNC with NIV as initial ventilation support strategy in AECOPD patients with respiratory acidosis. Propensity score matching (PSM) was implemented to increase between-group comparability. Kaplan–Meier analysis was utilized to evaluate differences between the HFNC success, HFNC failure, and NIV groups. Univariate analysis was performed to identify the features that differed significantly between the HFNC success and HFNC failure groups.

**Results:**

After screening 2219 hospitalization records, 44 patients from the HFNC group and 44 from the NIV group were successfully matched after PSM. The 30-day mortality (4.5% versus 6.8%, *p* = 0.645) and 90-day mortality (4.5% versus 11.4%, *p* = 0.237) did not differ between the HFNC and NIV groups. Length of ICU stay (median: 11 versus 18 days, *p* = 0.001), length of hospital stay (median: 14 versus 20 days, *p* = 0.001), and hospital cost (median: 4392 versus 8403 $USD, *p* = 0.001) were significantly lower in the HFNC group compared with NIV group. The treatment failure rate was much higher in the HFNC group than in the NIV group (38.6% versus 11.4%, *p* = 0.003). However, patients who experienced HFNC failure and switched to NIV showed similar clinical outcomes to those who first received NIV. Univariate analysis showed that log NT-proBNP was an important factor for HFNC failure (*p* = 0.007).

**Conclusions:**

Compared with NIV, HFNC followed by NIV as rescue therapy may be a viable initial ventilation support strategy for AECOPD patients with respiratory acidosis. NT-proBNP may be an important factor for HFNC failure in these patients. Further well-designed randomized controlled trials are needed for more accurate and reliable results.

## 1. Introduction

Chronic obstructive pulmonary disease is a common respiratory disease characterized by persistent respiratory symptoms and airflow limitation [1]. Patients may require hospitalization and respiratory support due to acute exacerbations. Noninvasive ventilation (NIV) is the first-line treatment for acute exacerbation of COPD (AECOPD) with respiratory acidosis [2]. However, up to 30% of AECOPD patients do not tolerate NIV due to interface discomfort, sputum retention, impaired communication, and facial skin breakdown [3, 4].

High-flow nasal cannula (HFNC) is a high-flow oxygen delivery system that can provide well-heated and humidified oxygen at continuous high flow rates up to 60 L/min via a large-bore nasal cannula [5]. Previous studies and literature reviews showed that HFNC had many beneficial effects in stable COPD patients, including a constant fraction of inspired oxygen delivery, dead space washout, improved comfort and tolerance, better communication, enhanced secretion clearance, and positive end-expiratory pressure (PEEP) effect, resulting in decreased PaCO_2_, reduced inspiratory effort, lower rate of moderate/severe exacerbations, and prolonged duration without exacerbations [6–13]. In AECOPD patients, recent studies also showed that HFNC can reduce PaCO_2_ and improve capillary pH as well as the work of breathing and patient comfort [14–16]. However, limited data are available about the clinical outcomes of AECOPD patients with respiratory acidosis using HFNC as initial ventilation support strategy compared with NIV [16–19].

The purpose of this study was to compare the effects of HFNC versus NIV as initiating ventilation support strategy on clinical outcomes in AECOPD patients with respiratory acidosis, as well as to explore the predictors of HFNC failure.

## 2. Methods

### 2.1. Study Design and Population

**Figure 1:**
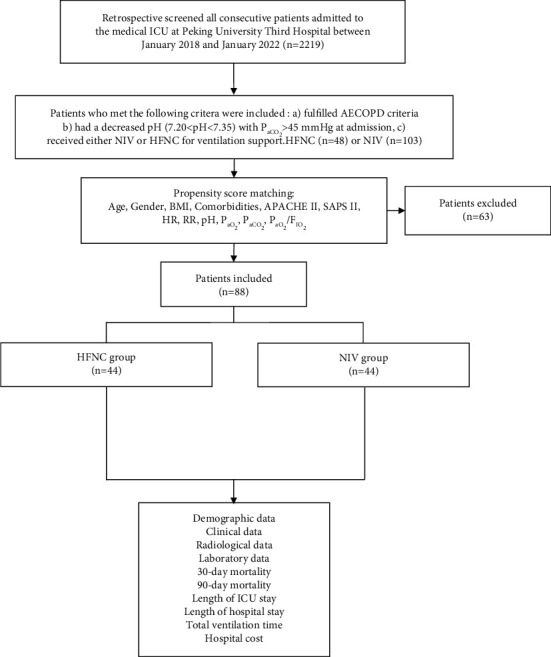
Patient selection diagram based on propensity score matching.

We reviewed all consecutive patients admitted to our 26-bed medical ICU between January 2018 and January 2022 at Peking University Third Hospital, a university-affiliated tertiary hospital in Beijing, China. AECOPD patients with respiratory acidosis were enrolled according to the following criteria: (a) fulfilled the AECOPD criteria [20], (b) had a decreased pH (7.20 < pH < 7.35) with PaCO_2_ > 45 mmHg upon admission, and (c) received either NIV or HFNC for initial ventilation support. Schematic of patient selection is shown in Figure 1. This study was approved by the Ethics Committee of Peking University Third Hospital (approval no. M2022265), and due to its observational nature, the requirement for informed consent was waived. All patient records and data were anonymized and deidentified prior to analysis.

### 2.2. Classification of Patients and Ventilation Settings

All the patients were treated with HFNC and NIV based on our ICU treatment protocol. Patients in the study were classified into two groups based on the ventilation support strategy they received: the HFNC group and the NIV group. The fraction of inspired oxygen was set to keep SpO_2_ at 88–92% or PaO_2_ at around 60 mmHg in both groups.

Patients in the HFNC group were treated with HFNC as initial ventilation support strategy and switched to NIV when the following criteria was met: (1) intolerance of nasal cannula or high flow rates; (2) worsened pH in conjunction with a rise in PaCO_2_ one hour after optimal flow rate settings (highest flow rates tolerated). HFNC was delivered by AIRVO-2™ (Fisher and Paykel Healthcare, Auckland, New Zealand). The initial flow rate was set at 25 L/min and gradually increased to the maximal tolerance of each patient [21]. When arterial blood gas was improved (pH > 7.35 with decreased PaCO_2_), the flow rate was gradually decreased (5 L/min each time) and HFNC was discontinued when the flow rate was less than 20 L/min. Treatment failure of HFNC was defined as switching to NIV. When patients in the HFNC group further deteriorated after switching to NIV and met the criteria for intubation, they would be intubated and receive invasive ventilation.

NIV was the initial ventilation support strategy in the NIV group. Noninvasive ventilation was applied with specified NIV ventilator V60 (Philips Respironics, California, United States) via a full-face mask. S/T mode was applied to all patients. Expiratory positive airway pressure (EPAP) was commenced at 4 H_2_O and titrated to diminish ineffective inspiratory efforts. Inspiratory positive airway pressure (IPAP) was set at 8 H_2_O and gradually increased to achieve a tidal volume of more than 6 mL/kg or to the maximum each patient could tolerate. IPAP was limited to no more than 25 H_2_O [22]. NIV was used as long as possible during the first 24 hours, until pH, PaCO_2_, and clinical condition improved. When arterial blood gas was improved (pH > 7.35 with decreased PaCO_2_), the duration of NIV was gradually decreased until the patient could sustain 24-hour spontaneous breathing without NIV [23]. NIV failure was defined as intubation or death during NIV.

The criteria for intubation in our department were in accordance with published literature, and intubation was left to the discretion of the physician [4, 22, 24]. Major criteria for intubation were (1) cardiac or respiratory arrest; (2) loss of consciousness; and (3) hemodynamic instability. Minor criteria were (1) unable to fit mask; (2) inability to protect the airway; (3) inability to clear secretions; (4) respiratory rate more than 35 breaths per minute; (5) signs of increased work of breathing, accessory muscle use, or abdominal paradox; and (6) worsened pH in conjunction with a rise in PaCO_2_ one hour after optimal ventilator settings (highest IPAP tolerated).

### 2.3. Data Collection

The following data were extracted from electronic medical records for all included patients: demographic information (age, gender, and body mass index), comorbidity severity scores including Charlson Comorbidity Index [25], Simplified Acute Physiology Score (SAPS) II, and Acute Physiology and Chronic Health Evaluation (APACHE) II scores, and outcomes, such as treatment failure, length of ICU stay, length of hospital stay, total ventilation time, and hospital cost. Additionally, clinical, radiological, and laboratory data on admission, such as heart rate, systolic blood pressure, respiratory rate, body temperature, echocardiography results, N-terminal prohormone, brain natriuretic peptide (NT-proBNP), FIO_2_, and arterial blood gas results, such as pH, PaO_2_, PaCO_2_, sodium bicarbonate, and PaO_2_-to-FIO_2_ ratio, were also collected. Settings of NIV or HFNC during the ICU stay were collected. A propensity score model was used to match patients, and clinical outcomes like 30-day mortality, 90-day mortality, treatment failure rate (defined by switching to NIV in the HFNC group, and intubation or death in the NIV group), length of ICU stay, length of hospital stay, total ventilation time, and hospital cost were compared after propensity score matching (PSM).

### 2.4. Statistical Analyses

Propensity score matching was applied to reduce the possibility of selection bias and confounding factors. Age, gender, BMI, APACHE II, SAPS II, comorbidities, heart rate, respiratory rate, pH, PaO_2_, PaCO2, and PaO_2_/FIO_2_ were included for propensity score matching. A multivariate logistic regression model was used to estimate patients' propensity score for receiving HFNC or NIV. A caliper of 0.15 was used for one-to-one nearest neighbor matching.

The consistency test of normal distribution for measurement data was carried out by the Shapiro–Wilk test. According to the distribution, continuous variables were reported as mean ± standard deviation (SD) or median (interquartile range (IQR), from 25^th^ to 75^th^ percentiles) and were compared with independent sample *t*-tests, Mann–Whitney *U* test, or Kruskal–Wallis test as appropriate. Categorical variables were expressed as percentages and were compared by Fisher's exact test or chi-square test when appropriate.

Univariate logistic analysis was performed to identify factors related to HFNC failure. Kaplan–Meier curves were drawn to assess the length of ICU stay, length of hospital stay, and total ventilation time, and log-rank tests were used to compare the differences between the HFNC failure, HFNC success, and NIV groups.

All statistical analyses were performed using SPSS 26.0 (IBM Corporation, Armonk, NY, USA). *p* values less than 0.05 were considered significant.

## 3. Results

### 3.1. Study Population

There were 2219 ICU admissions between January 2018 and January 2022, and 151 patients were included in this study, with 48 receiving HFNC and 103 receiving NIV. The baseline characteristics of both groups before propensity score matching are presented in Table 1. Compared with the HFNC group, patients in the NIV group exhibited lower GCS scores and a larger proportion of home NIV use, hypertension, and diabetes mellitus. After propensity score matching, there were 44 patients from the HFNC group and 44 patients from the NIV group, with mean ages of 78.4 and 80.2 years, respectively. The baseline characteristics of the two groups were well balanced after propensity score matching (Table 2). The ventilation settings of HFNC and NIV are shown in Table 3.

### 3.2. Clinical Outcomes

The clinical outcomes of the matched patients are shown in Table 4. The 30-day mortality (4.5% versus 6.8%, *p*=0.645), 90-day mortality (4.5% versus 11.4%, *p*=0.237), and intubation rate (4.5% versus 11.4%, *p*=0.237) did not differ between the HFNC and NIV groups. The treatment failure rate was significantly higher in the HFNC group than in the NIV group (38.6% versus 11.4%, *p*=0.003). However, length of ICU stay (11 (IQR, 7–15) versus 18 (IQR, 11–27) days, *p*=0.001), length of hospital stay (14 (IQR, 9–17) versus 20 (IQR, 16–30) days, *p*=0.001), total ventilation time (7 (IQR, 4–11) versus 13 (IQR, 7–23) days, *p*=0.001), and hospital cost (4392 (IQR, 3450–7889) versus 8403 (IQR, 5738–17469) $USD, *p*=0.001) were significantly lower in the HFNC group compared with NIV group.

**Figure 2:**
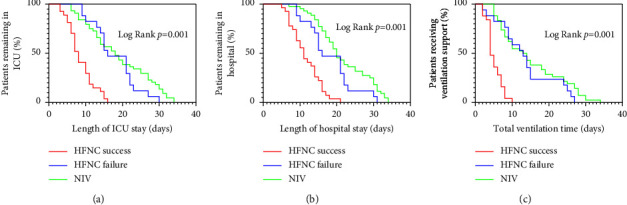
Kaplan–Meier curves for length of ICU stay (a), length of hospital stay (b), and total ventilation time (c) in HFNC success (red), HFNC failure (blue), and NIV (green) groups (log-rank test, *p*=0.001 for all).

We compared the differences in clinical outcomes between the HFNC success, HFNC failure, and NIV groups.  Figure 2 shows the Kaplan–Meier curves for length of ICU stay (Figure 2(a)), length of hospital stay (Figure 2(b)), and total ventilation time (Figure 2(c)). There was no significant difference between the HFNC failure group and the NIV group in terms of the length of ICU stay, length of hospital stay, or total ventilation time, while the HFNC success group showed a significantly lower result than the other two groups (Table 5).

In the HFNC group before PSM, 17 of 48 patients experienced treatment failure. We performed univariable logistic regression analyses to identify factors related to treatment failure in the HFNC group (Table 6). Univariate analysis showed that log-transformed NT-proBNP was an important factor for HFNC failure (*p*=0.007). However, due to the relative small number of patients who failed HFNC, the events per variable (EPV) were not adequate for multivariate logistic regression.

## 4. Discussion

The current study examined the impact of HFNC and NIV on clinical outcomes in AECOPD patients with respiratory acidosis admitted to the ICU. After screening and propensity score matching, 44 patients who received HFNC and 44 patients who received NIV were included in our study, with well-balanced baseline characteristics. Our current findings suggested that HFNC followed by NIV as rescue therapy might be a viable initiating ventilation strategy for AECOPD patients with respiratory acidosis, as there was no difference in 30-day mortality and 90-day mortality and significantly shorter length of ICU stay, length of hospital stay, and total ventilation time when compared to NIV therapy, which is the “gold standard” in these patients. In addition, NT-proBNP level upon admission was an important factor for HFNC failure.

The overall short-term mortality rate varies from 1.8% to 20.4% for hospitalized patients with AECOPD [26, 27]. Several clinical trials comparing HFNC with NIV in AECOPD patients with respiratory acidosis have indicated promising clinical outcomes with HFNC. In a prospective observational study, Lee and colleagues found no difference between the HFNC and NIV groups in terms of intubation rate (25% HFNC versus 27.3% NIV, *p*=0.857), 30-day mortality (15.9% HFNC versus 18.2% NIV, *p*=0.845), or device application days (7 HFNC versus 8 NIV, *p*=0.822) in patients with AECOPD and moderate hypercapnic respiratory failure [16]. In another observational cohort trial, Sun and colleagues compared the outcomes of HFNC and NIV in patients with COPD exacerbation and moderate acute hypercapnic respiratory failure (arterial pH between 7.25 and 7.35). Both groups had comparable device failure rates (28.2% HFNC versus 39.5% NIV, *p*=0.268) and 28-day mortality rates (15.4% HFNC versus 14% NIV, *p*=0.824). Neither the length of ICU stay (7 days HFNC versus 8 days NIV, *p*=0.149) nor hospital stay (9 days HFNC versus 10 days NIV, *p*=0.207) differed significantly between the two groups [18]. In a subgroup analysis of 65 patients from a large randomized controlled trial, Doshi and colleagues also found identical failure rates of HFNC and NIV at 72 h (23.5% versus 25.8%, *p*=1.000) [28]. A few randomized controlled trials also revealed similar clinical outcomes between the HFNC and NIV groups. In a randomized clinical trial, forty patients were randomly assigned to the HFNC group and NIV group. There was no difference in the duration of hospitalization (11.5 days HFNC versus 11.0 days NIV, *p*=0.655) or mortality rate (15% HFNC versus 15% NIV, *p*=0.669) between the two groups [19]. In a multicenter noninferiority randomized trial conducted by Cortegiani et al., eighty patients with mild-to-moderate AECOPD (arterial pH: 7.25–7.35, PaCO_2_ ≥ 55 mmHg before ventilator support) were randomly allocated to either the HFNC group or the NIV group. In terms of reducing PaCO_2_, both treatments showed a significant effect on PaCO_2_ reduction over time. After 2 h of treatment, HFNC was not statistically inferior to NIV since the mean difference in PaCO_2_ reduction between the two groups was 2.66 mmHg, which was within the noninferiority threshold of 10 mmHg. The length of hospital stay (10 days HFNC versus 13 days NIV, *p*=0.6579) and in-hospital mortality (5% HFNC versus 15.4% NIV, *p*=0.1543) were comparable across the two groups. In addition, 32% of patients in HFNC group switched to NIV by 6 hours, and 57.5% received NIV during hospitalization [17]. In a more recent large retrospective study of 173 hospitalized COPD patients receiving HFNC, 68 patients (39%) experienced HFNC failure [29].

In the present study, the 30-day and 90-day mortality rates of the overall cohort were 5.7% and 8.0%, respectively, which are consistent with the findings of previous studies [26, 27], reinforcing the external validation of our results. There was no difference in 30-day mortality between the HFNC and NIV groups, which is consistent with previous studies reporting mortality [16–19]. The mortality rate was comparable between one study (5% HFNC versus 15.4% NIV) and ours (4.5% HFNC versus 6.8% NIV) [17]. However, the 30-day mortality rate was significantly higher in the other three studies. In two studies, this may have been due to the overall lower PaO_2_/FIO_2_ in their studies compared with ours ((134.6 ± 7.4 mmHg versus 200.0 ± 53.4 mmHg) [16] and (139.2 ± 6.7 mmHg versus 200.0 ± 53.4 mmHg) [18]), indicating more severe respiratory failure. In the study conducted by Papachatzakis, the APACHE II scores of the enrolled patients were significantly higher than ours (20.5 versus 16), which represented a higher mortality risk. These factors may explain why our investigations yielded different outcomes.

In contrast to the results of previously mentioned studies [16–19], HFNC was substantially related to shorter length of ICU stay, hospital stay, and total ventilation days than NIV in this study. This result may be attributable to the longer length of ICU stay, hospital stay, and total ventilation days in the NIV group. High arterial PaCO_2_ and coexisting morbidities such as diabetes, hypertension, and cancer have been demonstrated to be related to extended ICU and hospital stays [30, 31]. In the present study, the patients enrolled had higher arterial PaCO_2_ and more coexisting morbidities, which may partially explain the discrepancy between the current study and earlier studies. Our results also showed that patients who responded well to HFNC may experience a significantly shorter length of total ventilation time, ICU stay, hospital stay, and lower hospital cost. Furthermore, when patients failed HFNC therapy and switched to NIV, clinical outcomes were no worse than NIV therapy alone (Figure 2).

In the present study, univariable logistic analysis showed that the NT-proBNP level upon admission might be an important factor for HFNC failure in AECOPD patients with respiratory acidosis. NT-proBNP is secreted by cardiac myocytes in response to increased arterial and ventricular filling pressure and is widely used for the diagnosis and management of heart failure [32, 33]. In addition to heart failure, NT-proBNP is also elevated in other conditions, including advanced age, renal failure, chronic lung disease, coronary heart disease, pulmonary hypertension, and sepsis [34]. Elevated NT-proBNP levels are also observed in AECOPD patients without primary cardiac abnormalities as a consequence of the release of NT-proBNP from the right ventricle caused by cor pulmonale, pulmonary hypertension, and hypoxemia [35]. Previous studies have shown that elevated NT-proBNP level is associated with worse in-hospital outcomes and is a reliable predictive biomarker of poor prognosis in patients with AECOPD [36, 37]. In a more recent study, Veenstra and colleagues found a significant association between cardiac (myocardial infarction, heart failure, or arrhythmia) (OR = 0.435, *p* = 0.013) and vascular (hypertension and peripheral arterial disease) (OR = 0.493, *p* = 0.035) comorbidity and a lower likelihood of HFNC success in AECOPD patients [29]. In the present study, our results further indicate that the NT-proBNP level upon admission might be an important factor for HFNC failure in AECOPD patients with respiratory acidosis.

Our study had several limitations. First, it was a retrospective observational study conducted at a single center, and there was a possibility of clinical selection bias regarding the ventilation support patients received. However, HFNC and NIV were both first-line options for these patients in our hospital, which could reduce selection bias. In addition, we used propensity score matching to balance the baseline characteristics between the two groups and evaluated the treatment outcomes based on the matched group, which could further reduce selection bias. Second, due to the relatively high age of patients in our study group, the latest pulmonary function test results were years before admission, which were not suitable to analyze the influence on clinical outcomes. Third, in the present study, we did not rule out patients with comorbid heart failure. As a result, heart failure accounted for 21.6% of the entire study population. NT-proBNP levels in the HFNC and NIV groups were 1251.0 (IQR, 367.0–2437.5) and 1995.0 (IQR, 510.3–3977.5) pg/ml, respectively. Considering the relatively normal LVEF (69.51 ± 6.10%) and high RVSP (38 (IQR, 28–50) mmHg), the elevation in NT-proBNP might be due to the complex interplay of heart failure with preserved ejection with right ventricular dysfunction. We were unable to further investigate the origin based on the retrospective data. Fourth, due to the relatively small number of patients who failed HFNC, the events per variable (EPV) were not adequate for multivariate logistic regression. So we could not further analyze whether NT-proBNP was an independent risk factor for HFNC failure or not. Finally, the sample size was relatively small due to propensity matching, and the results must be interpreted with caution. Large-scale, multicenter, and randomized controlled trials with larger sample sizes are still required to obtain more accurate and reliable results.

## 5. Conclusions

HFNC followed by NIV as rescue therapy may be a viable initial ventilation support strategy for AECOPD patients with respiratory acidosis, with lower hospital costs, shorter ICU and hospital stays, and similar clinical outcomes compared with NIV. NT-proBNP may be an important factor for HFNC failure in these patients. Further well-designed randomized controlled trials are needed to obtain more accurate and reliable results.

## Figures and Tables

**Table 1:** Baseline characteristics at the time of ICU admission before propensity score matching.

Characteristics	Unmatched cohort (*n* = 151)			
	Total (*n* = 151)	HFNC group (*n* = 48)	NIV group (*n* = 103)	*p* value
Demographics				
Age (years)	78.9 ± 8.3	78.6 ± 8.5	79.1 ± 8.3	0.762
Gender ((%) male)	68.9	75	66	0.267
BMI (kg/m^2^)	22.8 ± 5.6	22.9 ± 6.0	22.6 ± 5.2	0.804
Pack year	39.0 (15.0–45.0)	32.5 (10.0–50.0)	40.0 (20.0–45.0)	0.963
FEV1%	31.5 ± 12.3	31.6 ± 12.3	31.5 ± 12.6	0.990
FEV1/FVC	48.6 ± 10.7	49.6 ± 14.8	48.3 ± 9.6	0.764
Vital signs				
Body temperature (°C)	36.5 ± 0.5	36.6 ± 0.4	36.5 ± 0.5	0.195
Heart rate (beats/min)	89 ± 16	89 ± 13	90 ± 17	0.815
Respiratory rate (breaths/min)	22 ± 5	23 ± 4	22 ± 6	0.363
Systolic blood pressure (mmHg)	131 ± 21	129 ± 22	132 ± 21	0.479
Mean arterial pressure (mmHg)	89 ± 13	88 ± 12	91 ± 4	0.195
Severity score				
Charlson	6 ± 2	6 ± 2	6 ± 2	0.104
APACHE II	16 ± 4	16 ± 4	16 ± 4	0.526
SAPS II	33 ± 7	32 ± 5	34 ± 7	0.209
GCS	15 ± 2	15 ± 1	14 ± 2	0.032
Echocardiography				
LVEF (%)	68.8 ± 7.7	70.1 ± 4.8	68.2 ± 8.7	0.155
LVEDD (mm)	44.2 ± 6.4	43.9 ± 5.2	44.5 ± 6.9	0.584
RVEDD (mm)	22.1 ± 5.4	23.2 ± 6.7	22.0 ± 5.0	0.241
RVSP (mmHg)	38 (25–51)	39 (28–53)	36 (22–50)	0.375
Home NIV use no. (%)	34 (22.7)	5 (10.4)	29 (28.4)	0.014
Comorbidities, no. (%)				
Pneumonia	28 (18.7)	9 (18.8)	19 (18.6)	0.986
Cor pulmonale	41 (27.3)	14 (29.2)	27 (26.5)	0.730
Heart failure	34 (22.7)	8 (16.7)	26 (25.5)	0.229
Hypertension	117 (78.0)	32 (66.7)	85 (83.3)	0.022
Coronary artery disease	40 (26.7)	8 (16.7)	32 (31.4)	0.057
Old myocardial infarction	18 (12)	4 (8.3)	14 (13.7)	0.343
Atrial fibrillation	33 (22.0)	10 (20.8)	23 (22.5)	0.813
OSAHS	7 (4.7)	3 (6.3)	4 (3.9)	0.681
Diabetes mellitus	39 (26)	5 (10.4)	34 (33.3)	0.003
Arterial blood gas				
pH	7.34 ± 0.08	7.33 ± 0.07	7.34 ± 0.09	0.878
PaCO_2_ (mmHg)	69.6 ± 20.7	68.3 ± 20.8	70.3 ± 20.7	0.601
HCO_3−_ (mmol/L)	36.6 ± 8.3	35.6 ± 7.4	37.1 ± 8.7	0.299
PaO_2_/FIO_2_ (mmHg)	206.4 ± 54.4	203.5 ± 55.6	207.7 ± 54.3	0.681
Laboratory parameters				
White blood cell (×10^9^)	9.36 (7.08–12.86)	8.73 (6.54–12.18)	9.84 (7.24–12.94)	0.304
Neutrophil (%)	80.3 ± 9.0	79.1 ± 9.3	80.9 ± 8.8	0.261
PCT (*μ*g/L)	0.11 (0.10–0.20)	0.10 (0.10–0.15)	0.12 (0.10–0.25)	0.038
CRP (mg/L)	2.69 (1.00–8.20)	3.67 (1.10–8.43)	2.43 (0.91–8.65)	0.598
Albumin (g/L)	37.2 ± 5.3	36.3 ± 5.0	37.7 ± 5.4	0.126
Creatinine (*μ*mol/L)	66.0 (55.0–87.0)	70.0 (59.3–90.0)	63.0 (55.0–87.0)	0.315
CCR (mL/min)	60.7 (48.9–82.1)	58.5 (52.2–69.0)	63.0 (45.6–86.9)	0.499
NT-proBNP (pg/dl)	1460.0 (411.0–3510.0)	1196.5 (367.0–2437.5)	1710.0 (472.0–3930.0)	0.123

Data are presented as mean ± standard deviation, median (interquartile range), no. (%), or %. APACHE, Acute Physiology and Chronic Health Evaluation; BMI, body mass index; CCR, creatinine clearance rate; CRP, C-reactive protein; FEV 1, forced expiratory volume in one second; FVC, forced vital capacity; GCS, Glasgow Coma Scale; HFNC, high-flow nasal cannula; ICU, intensive care unit; LVEDD, left ventricle end-diastolic diameter; LVEF, left ventricular ejection fraction; NIV, noninvasive ventilation; NT-proBNP, N-terminal pro-brain natriuretic peptide; OSAHS, obstructive sleep apnea and hypoventilation syndrome; RVEDD, right ventricle end-diastolic diameter; RVSP, right ventricular systolic pressure; PCT, procalcitonin; SAPS, Simplified Acute Physiology Score.

**Table 2:** Baseline characteristics at the time of ICU admission after propensity score matching.

Characteristics	Matched cohort (*n* = 88)			
	Total (*n* = 88)	HFNC group (*n* = 44)	NIV group (*n* = 44)	*p* value
Demographics				
Age (years)	79.3 ± 8.5	78.4 ± 8.7	80.2 ± 8.3	0.324
Gender ((%) male)	73.9	75.0	72.7	0.808
BMI (kg/m^2^)	22.5 ± 5.6	22.5 ± 6.1	22.4 ± 5.0	0.936
Pack year	37.5 (10.6–50.0)	32.5 (10.0–57.5)	40.0 (15.6–45.0)	0.883
FEV1%	32.4 ± 12.0	28.7 ± 10.5	34.9 ± 12.8	0.344
FEV1/FVC	48.23 ± 12.19	47.27 ± 14.29	48.99 ± 11.14	0.790
Vital signs				
Body temperature (°C)	36.6 ± 0.5	36.6 ± 0.5	36.5 ± 0.5	0.571
Heart rate (beats/min)	89 ± 15	89 ± 13	88 ± 17	0.627
Respiratory rate (breaths/min)	23 ± 5	23 ± 4	22 ± 5	0.723
Systolic blood pressure (mmHg)	131 ± 20	128 ± 21	133 ± 19	0.291
Mean arterial pressure (mmHg)	89 ± 13	87 ± 12	90 ± 14	0.286
Severity score				
Charlson	6 ± 2	6 ± 2	7 ± 2	0.195
APACHE II	16 ± 4	16 ± 4	17 ± 4	0.591
SAPS II	33 ± 6	32 ± 5	34 ± 7	0.132
GCS	15 ± 1	15 ± 1	15 ± 1	0.267
Echocardiography				
LVEF (%)	69.5 ± 6.1	70.4 ± 4.8	68.7 ± 7.1	0.191
LVEDD (mm)	44.1 ± 6.1	44.0 ± 5.3	44.2 ± 6.9	0.905
RVEDD (mm)	22.9 ± 6.2	23.4 ± 6.8	22.3 ± 5.7	0.446
RVSP (mmHg)	38 (28–51)	38 (28–50)	39 (27–51)	0.986
Home NIV use no. (%)	14 (15.9)	5 (11.4)	9 (20.5)	0.244
Comorbidities, no. (%)				
Pneumonia	17 (19.3)	8 (18.2)	9 (20.5)	0.787
Cor pulmonale	23 (26.1)	13 (29.5)	10 (22.7)	0.467
Heart failure	19 (21.6)	8 (18.2)	11 (25.0)	0.437
Hypertension	71 (81.7)	34 (77.3)	37 (84.1)	0.418
Coronary artery disease	19 (21.6)	8 (18.2)	11 (25.0)	0.437
Old myocardial infarction	10 (11.4)	4 (9.1)	6 (13.6)	0.502
Atrial fibrillation	21 (23.9)	10 (22.7)	11 (25.0)	0.803
OSAHS	7 (8.0)	3 (6.8)	4 (9.1)	0.694
Diabetes mellitus	13 (14.8)	5 (11.4)	8 (18.2)	0.367
Arterial blood gas				
pH	7.33 ± 0.08	7.33 ± 0.06	7.33 ± 0.10	0.900
PaCO_2_ (mmHg)	70.1 ± 19.5	70.2 ± 19.7	70.0 ± 19.4	0.952
HCO_3−_ (mmol/L)	36.35 ± 7.63	36.20 ± 7.15	36.50 ± 8.18	0.859
PaO_2_/FIO_2_ (mmHg)	200.0 ± 53.4	197.3 ± 58.7	202.6 ± 48.2	0.645
Laboratory parameters				
White blood cell (×10^9^)	9.25 (6.05–12.91)	8.62 (6.06–12.11)	10.81 (6.05–13.56)	0.280
Neutrophil (%)	80.6 ± 9.5	78.9 ± 9.4	82.3 ± 9.4	0.093
PCT (*μ*g/L)	0.11 (0.10–0.17)	0.10 (0.10–0.15)	0.13 (0.10–0.22)	0.124
CRP (mg/L)	2.70 (1.17–8.63)	2.70 (1.03–8.63)	3.11 (1.27–8.62)	0.932
Albumin (g/L)	37.0 ± 5.1	36.2 ± 4.7	37.7 ± 5.4	0.166
Creatinine (*μ*mol/L)	66.5 (55.0–88.8)	70.0 (59.3–89.5)	62.0 (54.3–87.3)	0.411
CCR (mL/min)	58.8 (48.9–77.0)	57.9 (51.3–69.0)	60.3 (45.6–87.2)	0.717
NT-proBNP (pg/dl)	1530.0 (409.5–3485.0)	1251.0 (367.0–2437.5)	1995.0 (510.3–3977.5)	0.058

Data are presented as mean ± standard deviation, median (interquartile range), no. (%), or %. APACHE, Acute Physiology and Chronic Health Evaluation; BMI, body mass index; CCR, creatinine clearance rate; CRP, C-reactive protein; FEV 1, forced expiratory volume in one second; FVC, forced vital capacity; GCS, Glasgow Coma Scale; HFNC, high-flow nasal cannula; ICU, intensive care unit; LVEDD, left ventricle end-diastolic diameter; LVEF, left ventricular ejection fraction; NIV, noninvasive ventilation; NT-proBNP, N-terminal pro-brain natriuretic peptide; OSAHS, obstructive sleep apnea and hypoventilation syndrome; RVEDD, right ventricle end-diastolic diameter; RVSP, right ventricular systolic pressure; PCT, procalcitonin; SAPS, Simplified Acute Physiology Score.

**Table 3:** Ventilation settings of HFNC and NIV.

	HFNC group (*n* = 44)	NIV group (*n* = 44)	*p* value
Flow rate (L/min)	48.3 ± 8.6	—	—
IPAP ((cm) H_2_O)	—	19.0 ± 3.2	—
EPAP ((cm) H_2_O)	—	6.3 ± 1.8	—
FIO_2_ (%)	39.9 ± 0.2	40.7 ± 0.1	0.796

Data are presented as mean ± standard deviation. EPAP, expiratory positive airway pressure; IPAP, inspiratory positive airway pressure.

**Table 4:** Clinical outcomes after propensity score matching.

Variables	Total (*n* = 88)	HFNC (*n* = 44)	NIV group (*n* = 44)	*p* value
30-day mortality (%)	5 (5.7)	2 (4.5)	3 (6.8)	0.645
90-day mortality (%)	7 (8.0)	2 (4.5)	5 (11.4)	0.237
Treatment failure (%)	22 (25)	17 (38.6)	5 (11.4)	0.003
Intubation (%)	7 (8.0)	2 (4.5)	5 (11.4)	0.237
Total ventilation time (days)	8 (5–15)	7 (4–11)	13 (7–23)	0.001
Length of ICU stay (days)	14 (8–22)	11 (7–15)	18 (11–27)	0.001
Length of hospital stay (days)	16 (12–22)	14 (9–17)	20 (16–30)	0.001
Hospital cost ($USD)	6448 (4124–11013)	4392 (3450–7889)	8403 (5738–17469)	0.001

Data are presented as median (interquartile range) or no. (%).

**Table 5:** Clinical outcomes of HFNC success, HFNC failure, and NIV groups.

Variables	HFNC (*n* = 44)		NIV group (*n* = 44)	*p* value
	Success group (*n* = 27)	Failure group (*n* = 17)		
30-day mortality (%)	0 (0)	2 (11.8)	3 (6.8)	0.234
90-day mortality (%)	0 (0)	2 (11.8)	5 (11.4)	0.186
Intubation (%)	0 (0)	2 (11.8)	5 (11.4)	0.186
Total ventilation time (days)	5 (4–7)	13 (9–19)	13 (7–23)	0.001
Length of ICU stay (days)	8 (6–11)	16 (13–22)	18 (11–27)	0.001
Length of hospital stay (days)	11 (8–15)	16 (14–22)	20 (16–30)	0.001
Hospital cost ($USD)	3854 (2976–5377)	7619 (4049–11002)	8403 (5738–17469)	0.001

Data are presented as median (interquartile range) or no. (%).

**Table 6:** Univariate logistic regression analysis of factors related to HFNC failure.

Variable	Univariate analysis	
	OR (95% CI)	*p* value
Gender	1.429 (0.374–5.459)	0.602
Age (years)	1.077 (0.989–1.174)	0.088
Body mass index	1.084 (0.975–1.205)	0.135
Charlson	1.362 (0.979–1.895)	0.103
Home NIV use	9.231 (0.939–90.781)	0.057
Body temperature (°C)	1.816 (0.477–6.915)	0.382
Hear rate (beats/min)	0.987 (0.942–1.033)	0.567
Respiratory rate (breaths/min)	1.095 (0.943–1.270)	0.233
Mean arterial pressure (mmHg)	0.958 (0.906–1.013)	0.129
APACHE II	1.064 (0.903–1.253)	0.459
GCS	0.001 (0)	0.999
SAPS II	1.114 (0.982–1.264)	0.102
White blood cell (10^9^)	0.940 (0.821–1.077)	0.375
Neutrophil (%)	0.977 (0.916–1.043)	0.489
CRP (mg/L)	0.925 (0.691–1.238)	0.599
PCT (*μ*g/L)	0.933 (0.645–1.350)	0.713
Log NT-proBNP (pg/dl)	7.506 (1.746–32.263)	0.007
Creatinine (*μ*mol/L)	1.012 (0.997–1.027)	0.128
CCR (mL/min)	0.996 (0.963–1.031)	0.835
Albumin (g/L)	0.950 (0.840–1.075)	0.417
LVEF (%)	1.104 (0.965–1.264)	0.148
RVSP (mmHg)	0.966 (0.967–1.027)	0.808
pH on admission	0.001 (0.000–20.933)	0.170
PaCO_2_ on admission	1.021 (0.989–1.053)	0.201
PaO_2_ on admission	0.991 (0.969–1.013)	0.409
PaO_2_/FIO_2_ on admission	0.988 (0.975–1.002)	0.101
Pneumonia	0.893 (0.193–4.134)	0.885
Cor pulmonale	1.019 (0.278–3.737)	0.978
Heart failure	3.611 (0.739–17.644)	0.113
Hypertension	0.391 (0.112–1.361)	0.140
Coronary artery disease	2.167 (0.526–8.926)	0.284
Old myocardial infarction	1.429 (0.374–5.459)	0.602
Atrial fibrillation	1.077 (0.989–1.174)	0.088
OSAHS	4.000 (0.335–47.726)	0.273
Diabetes mellitus	9.231 (0.939–90.781)	0.057

APACHE, Acute Physiology and Chronic Health Evaluation; CCR, creatinine clearance rate; CRP, C-reactive protein; GCS, Glasgow Coma Scale; HFNC, high-flow nasal cannula; ICU, intensive care unit; LVEF, left ventricular ejection fraction; Log NT-proBNP, log-transformed N-terminal pro-brain natriuretic peptide; OSAHS, obstructive sleep apnea and hypoventilation syndrome; PCT, procalcitonin; RVSP, right ventricular systolic pressure; SAPS, Simplified Acute Physiology Score.

## Data Availability

The datasets analyzed during this study are available from the corresponding author upon reasonable request.
